# Cardiac involvement in hospitalized patients with COVID-19 and its incremental value in outcomes prediction

**DOI:** 10.1038/s41598-021-98773-4

**Published:** 2021-09-30

**Authors:** Payam Pournazari, Alison L. Spangler, Fawzi Ameer, Kobina K. Hagan, Mauricio E. Tano, Mohammed Chamsi-Pasha, Lakshmi H. Chebrolu, William A. Zoghbi, Khurram Nasir, Sherif F. Nagueh

**Affiliations:** 1 Houston Methodist DeBakey Heart and Vascular Center, Houston, USA; 2 Houston Methodist DeBakey Heart and Vascular Center, 6550 Fannin St, Suite 1800, Houston, TX 77030 USA

**Keywords:** Cardiology, Cardiovascular biology

## Abstract

Recent reports linked acute COVID-19 infection in hospitalized patients to cardiac abnormalities. Studies have not evaluated presence of abnormal cardiac structure and function before scanning in setting of COVD-19 infection. We sought to examine cardiac abnormalities in consecutive group of patients with acute COVID-19 infection according to the presence or absence of cardiac disease based on review of health records and cardiovascular imaging studies. We looked at independent contribution of imaging findings to clinical outcomes. After excluding patients with previous left ventricular (LV) systolic dysfunction (global and/or segmental), 724 patients were included. Machine learning identified predictors of in-hospital mortality and in-hospital mortality + ECMO. In patients without previous cardiovascular disease, LV EF < 50% occurred in 3.4%, abnormal LV global longitudinal strain (< 16%) in 24%, and diastolic dysfunction in 20%. Right ventricular systolic dysfunction (RV free wall strain < 20%) was noted in 18%. Moderate and large pericardial effusion were uncommon with an incidence of 0.4% for each category. Forty patients received ECMO support, and 79 died (10.9%). A stepwise increase in AUC was observed with addition of vital signs and laboratory measurements to baseline clinical characteristics, and a further significant increase (AUC 0.91) was observed when echocardiographic measurements were added. The performance of an optimized prediction model was similar to the model including baseline characteristics + vital signs and laboratory results + echocardiographic measurements.

## Introduction

Several recent reports linked COVID-19 infection to cardiac abnormalities as determined by echocardiography and CMR^1–23^. The presence of worse right ventricular (RV) function was shown to be associated with worse outcomes, albeit likely related to hypoxemia and pulmonary vascular abnormalities as opposed to primary cardiac disease. Interestingly, there are data showing in early disease, cardiac index is increased and systemic vascular resistance is decreased with normal RV function^24^.

Notwithstanding the above findings, most studies have not evaluated the presence of abnormal cardiac structure and function before imaging in the setting of COVD-19 infection which could have led to an apparently higher incidence of cardiac abnormalities in the setting of COVID-19 infection. In some studies, data were based on a completed survey questionnaire, confounding the interpretation of the results. Further, it is important to study the incremental prognostic value of new onset cardiac disease over clinical and laboratory data which are routinely available in the setting of acute COVID-19 infection to determine the clinical relevance of new onset cardiac abnormalities. We therefore sought to examine cardiac function in a consecutive group of patients hospitalized with acute COVID-19 infection according to the presence or absence of cardiac disease based on electronic health records review, including cardiovascular imaging studies. We also looked at the independent contribution of echocardiographic findings to clinical outcomes.

## Methods

Consecutive patients between March 2020 and September 2020 who were hospitalized with COVID-19 diagnosis, and who underwent at least one echocardiogram were enrolled *in this retrospective study*. No patients were excluded a priori for suboptimal echocardiographic imaging. Diagnostic testing and management were determined by the treating physicians based on clinical status. Medical records were reviewed to determine the presence of CAD (coronary artery disease), heart failure, comorbid conditions, laboratory measurements, and clinical outcomes. Data were obtained from Houston Methodist’s (HM) COVID-19 Surveillance and Outcomes Registry (CURATOR), after Houston Methodist Research Institute IRB approval was granted. CURATOR is an Institutional Review Board (IRB) approved registry of socio-demographic, clinical, and outcomes data abstracted from the electronic medical records of individuals with COVID-19-related encounters within the HM system. CURATOR, through a secure HIPAA-compliant server houses COVID-19-related data from March 2020. Clinical records dating back to June 2016 are included in the database review. These records include, among others, demographics, laboratories, procedures, and results of imaging tests. For individuals with multiple hospitalizations, only first-time hospitalization was included in the current analysis. All COVID-19 cases were confirmed by an antigen test, reverse transcriptase-polymerase chain reaction (RT-PCR) test, or SARS-CoV-2 serology.

All methods were carried out in accordance with Houston Methodist Research Institute guidelines and regulations. Houston Methodist Research Institute IRB granted waiver of informed consent (45 CFR 46.116) when it approved the study. There were no experimental protocols examined in this study.

### Echocardiographic imaging

The echocardiographic study followed standard guidelines^25^. The indications for echocardiography were shortness of breath, positive troponin assays, and chest pain. Image acquisition was carried out at a frame rate of 40–80 frames/s, and 3 cardiac cycles were acquired in cine loop format. Studies were analyzed offline. From the apical window, PW (pulse wave) Doppler was used to record mitral inflow for 3–5 cardiac cycles at the level of the mitral valve annulus and tips^26^. Pulmonary venous flow was acquired in the apical 4 chamber view with the aid of color Doppler. A 2–3 mm sample volume was placed > 0.5 cm from LA (left atrium/left atrial) junction into the pulmonary vein^26^. Tissue Doppler (TD) was applied to record mitral annular velocities at the septal and lateral sides of mitral annulus, and lateral side of the tricuspid annulus^25,26^. The resulting annular velocities by PW Doppler were recorded for 5 cardiac cycles at a sweep speed of 100 mm/s. The tricuspid regurgitation velocity was recorded by CW (continuous wave) Doppler from multiple windows, and the highest velocity was noted. Imaging of the inferior vena cava and hepatic venous flow was obtained. Depending on the image quality, an ultrasound enhancing agent (UAE) was injected intravenously, if needed.

### Echocardiographic analysis

Echocardiographic measurements were performed by an observer without knowledge of clinical status. LV (left ventricular/left ventricle) volumes, mass, EF (ejection fraction), and left atrial volumes were measured per ASE (American Society of Echocardiography) guidelines^25^. Mitral inflow from the tips level was analyzed for peak early (E), and late (A) diastolic velocities, E/A ratio, and deceleration time (DT) of mitral E velocity^26^. Mitral annulus early (eʹ) diastolic velocity was measured at septal and lateral mitral annulus, and septal, lateral and average E/eʹ ratios were computed^26^. If satisfactory acquisition was achieved, pulmonary venous flow was evaluated for systolic to diastolic velocity ratio (used in patients with reduced LV EF as a marker of elevated left atrial pressure), and atrial velocity as an indicator of LV end diastolic pressure. The tricuspid regurgitation velocity was used along with inferior vena cava size and collapse and hepatic venous flow to estimate pulmonary artery (PA) systolic pressure based on the modified Bernoulli equation^25,26^. Measurements were averaged over 3 cardiac cycles. Tricuspid annular plane systolic excursion (TAPSE) and tricuspid sʹ (systolic ejection) velocity were determined^25^. LV global longitudinal strain (GLS) from the 3 apical views, RV free wall strain, and LA strain in apical 4-chamber view were measured by an observer blinded to clinical data and all other echocardiographic measurements^25,27^.

### Independent variables and outcomes

#### Independent variables

Biographic data (age, sex, race, ethnicity) were obtained. Patient medical histories were queried for the presence of pre-existing conditions, including obesity, heart disease, diabetes, respiratory disease, and renal disease, via International Classification of Disease (ICD)-10 codes, Diagnosis-related group (DRG) codes, with verification by chart review. These variables were grouped into patients’ baseline characteristics set.

For index hospitalization, vital signs at admission and during echocardiographic scanning, and laboratory parameters were tabulated. Vital signs obtained were heart rate, respiratory rate, arterial oxygen saturation, and systolic and diastolic blood pressure. Laboratory parameters included B-natriuretic peptide (BNP), troponin, C-reactive protein (CrP), interleukin-6 (IL-6), ferritin, aspartate aminotransferase (AST), alanine aminotransferase (ALT), lactate dehydrogenase (LDH), serum creatinine, venous pH, lactic acid, and partial pressure of oxygen in arterial blood (PaO_2_). These variables were grouped into laboratory and vital signs data set.

Left ventricular (LV) end-diastolic, end-systolic volumes, mass, and LA and RA (right atrial) maximum volumes were included in statistical analysis after indexing to BSA. LV diastolic function was determined based on guidelines^26^ with the following categories: normal, grade I, grade II, grade III, and indeterminate. For right ventricular systolic function, tricuspid annular plane systolic excursion (TAPSE) by M-mode imaging and tricuspid lateral annular systolic velocity (*S’*) using tissue Doppler imaging from the apical 4-chamber view were noted. Tricuspid (TR) and mitral regurgitation (MR) were categorized as none-trace, mild, mild-moderate, and moderate-severe lesions^28^. LV global longitudinal strain (GLS), RV free wall and total strain, and LA reservoir, conduit, and pump strain were also included in the echocardiographic variables data set.

### COVID-19 care indicators and hospital outcomes

Outcomes included in-hospital mortality, intensive care unit (ICU) admission, mechanical ventilation, and extracorporeal membrane oxygenation (ECMO) use. The primary outcome of interest was in-hospital death. Secondary outcome was in-hospital mortality and ECMO use.

### Statistical analysis

Analyses were done using Stata (v.16 STAT Corp Austin, TX) and Python. All continuous variables were tested for normality of their distributions. The study sample’s characteristics were summarized using mean (± standard deviation), median (interquartile range) and proportions. Two-group comparisons of these characteristics were conducted using χ^2^-test or Fisher’s exact test for categorical variables, two-sample t-test or Mann–Whitney U test for continuous variables depending on the normality of their distributions.

Machine learning was used to identify predictors of in-hospital mortality. Mean replacement was applied for missing data. Continuous variables were standardized using mean and standard deviation of respective variables. Three multivariable logistic regression models were fitted using in-hospital mortality as the outcome variable. In the first model, independent variables in the patients’ characteristics set were entered. In the second one, patient baseline characteristics and laboratory and vital signs data sets were included. In the third model, patients’ baseline characteristics, laboratory and vital signs parameters, and echocardiographic measurements were included. The three models were compared with respect to the area under the receiver operator curve (AUC).

Finally, an optimized prediction model for in-hospital mortality was developed by iteratively adding variables one-by-one into a logistic regression model regarding their influence in increasing the AUC in a hierarchical manner. In this process, at first, the AUCs for models fitted with one independent variable was evaluated. All variables in the three sets were tested. Then, the variable that showed the largest AUC was retained and added into the logistic model. Then, all remaining variables were added one by one to the model and the AUCs were computed. The variable with the largest AUC was selected and added to the logistic model. The procedure was repeated until the AUC augmented $<{10}^{-4}$ with the addition of new variables. The final model was compared against the three logistic regression models.

## Results

Initially, there were 768 patients for analysis. After excluding 44 patients with echocardiograms showing systolic dysfunction, and patients on mechanical circulatory support including LV assist devices prior to COVID diagnosis, 724 patients were included in the study. Table 1 shows a summary of clinical characteristics according to the presence or absence of cardiac disease. Patients with a previous diagnosis of cardiac disease (186 patients, 25.7%) were significantly older and had higher prevalence of chronic renal disease (both P < 0.001). Table 2 presents a summary of vital signs and laboratory measurements. BNP and troponin levels were significantly higher in patients with previous cardiac diagnosis in comparison with those without previous cardiac diagnosis (both P < 0.001).

**Table 1:** Baseline characteristics of the sample of adults hospitalized for COVID-19 stratified by prior cardiac diagnosis.

Variables	Total	No prior cardiac diagnosis	Prior cardiac diagnosis	P-value
	724	538 (74.2)	186 (25.7)	
Age, years	60.9 (16.5)	57.6 (16.7)	68.9 (13.1)	< 0.001
**Age group, years**				< 0.001
18–44	117 (16.2)	104 (19.4)	13 (7.0)	
45–64	118 (16.3)	101 (18.8)	17 (9.1)	
55–64	182 (25.1)	147 (27.4)	35 (18.8)	
65–74	148 (20.3)	103 (19.0)	45 (24.2)	
≥ 75	159 (22.0)	83 (15.5)	76 (40.9)	
**Sex**				0.017
Male	374 (51.5)	274 (51.0)	100 (53.2)	
Female	350 (48.3)	263 (49.0)	87 (46.8)	
**Race/ethnicity**				< 0.001
Non-Hispanic White	186 (25.4)	112 (20.9)	74 (38.8)	
Non-Hispanic Black	200 (27.6)	142 (26.4)	58 (31.2)	
Non-Hispanic Asian	28 (3.9)	23 (4.3)	5 (2.7)	
Non-Hispanic other	15 (2.1)	12 (2.2)	3 (1.6)	
Hispanic	277 (38.3)	233 (43.4)	44 (23.7)	
Body mass index, kg/m^2^	31.63 ± 8.31	32.1 ± 8.12	30.22 ± 8.70)	< 0.001
Obesity (BMI ≥ 30 kg/m^2^)	351 (48.5)	279 (52.0)	72 (38.7)	0.002
**Comorbidities**				
Diabetes mellitus	374 (51.7)	277 (51.6)	97 (52.2)	0.894
Renal disease	488 (67.4)	330 (61.5)	158 (85.0)	< 0.001

Mean ± standard deviation and number (column%) presented.

**Table 2:** Summary of vital signs and laboratory markers at index COVID-19 hospitalization stratified by prior cardiac diagnosis.

Variables	Total	No prior cardiac diagnosis	Prior cardiac diagnosis	P-value
	724	538 (74.2)	186 (25.7)	
**Vital signs at admission**				
Respiratory rate, breaths/min	21.92 ± 7.83	22.13 ± 8.21	21.30 ± 6.57	0.383
Pulse rate, beats/min	80.69 ± 18.16	80.42 ± 17.28	81.48 ± 20.53	0.747
Systolic pressure	127.88 ± 23.35	127.89 ± 22.73	127.87 ± 25.11	0.658
Diastolic pressure	67.49 ± 15.23	67.97 ± 15.83	66.12 ± 13.30	0.034
Oxygen saturation	95.86 ± 4.59	95.91 ± 4.54	95.71 ± 4.74	0.691
**Laboratory markers**				
Partial pressure of oxygen, mmHg	86 (68–118)	86 (69–117)	90 (64–123)	0.799
BNP, pg/ml	57 (16–165)	37 (11–103)	180 (72–494)	< 0.001
Troponin, ng/ml	0.02 (0.01–0.08)	0.01 (0.01–0.06)	0.04 (0.01–0.10)	< 0.001
C-reactive protein, mg/dl	7.08 (2.45–14.77)	6.85 (2.40–15.64)	7.31 (2.72–14.22)	0.927
Interleukin-6, pg/ml	51.3 (15–205)	51 (13.7–211)	53 (20–182)	0.556
Ferritin, ng/ml	780 (360.5–1454.5)	790 (375–1441)	720 (282–1515)	0.381
Alkaline phosphatase, U/l	75 (60–102)	75 (60–102)	75 (59.5–101)	0.939
Alanine transaminase, U/l	30 (19–54)	33 (20–56)	23.5 (16–40)	< 0.001
Aspartate transaminase, U/l	41 (27–63)	41 (28–63)	38 (23–62)	0.239
Lactate dehydrogenase, U/l	357.5 (253.5–494)	363 (254–499)	333 (252–475)	0.240
Serum creatinine, mg/dl	0.97 (0.73–1.49)	0.92 (0.70–1.29)	1.14 (0.87–1.97)	< 0.001
Venous pH	7.39 (7.34–7.42)	7.39 (7.34–7.43)	7.37 (7.32–7.41)	0.071
Lactic acid, mmol/l	1.80 (1.30–2.40)	1.80 (1.30–2.40)	1.70 (1.30–2.50)	0.805

Mean ± standard deviation or median (interquartile range) presented.

*BNP* B-natriuretic peptide.

### Echocardiographic findings

Table 3 presents a summary of the echocardiographic findings in the study sample. None of the patients had aortic stenosis or more than mild aortic regurgitation. None of the patients had more than mild mitral regurgitation. Most patients without prior cardiac disease did not have pericardial effusion, though 9.5% had a mild effusion, 0.4% had moderate effusion, and another 0.4% had large effusion. The majority of patients without prior cardiac disease had normal LV GLS (76%), normal tricuspid sʹ velocity (88%), and normal RV free wall strain (84%). In patients without prior cardiac disease, LV diastolic function was normal in 75%, grade I in 14%, grade II or III in 6%, and indeterminate in the remaining patients. Expectedly, patients with history of prior cardiac disease had a significantly higher incidence of grade I diastolic dysfunction at 20%, and grades II and III diastolic dysfunction at 31% (P < 0.001 vs patients without prior cardiac disease).

**Table 3:** Summary of echocardiographic findings by prior cardiac diagnosis.

Variables	Total	No prior cardiac diagnosis	Prior cardiac diagnosis	P-value
	724	538 (74.2)	186 (25.7)	
Left ventricular end-diastolic volume index	56.41 ± 17.01	55.36 ± 16.15	59.66 ± 19.10	0.025
Left ventricular end-systolic volume index	20.51 ± 9.87	19.46 ± 8.94	23.74 ± 11.78	< 0.001
Stroke volume index	35.84 ± 10.67	35.86 ± 10.40	35.79 ± 11.49	0.747
Ejection fraction,	64.04 ± 9.82	65.11 ± 8.85	60.90 ± 11.70	< 0.001
Left atrial volume index	30.16 ± 14.67	27.42 ± 11.26	38.70 ± 19.91	< 0.001
Right atrial volume index	25.46 ± 15.43	22.53 ± 11.87	34.09 ± 20.67	< 0.001
Left ventricular mass index	82.86 ± 25.57	79.63 ± 23.15	92.18 ± 29.70	< 0.001
Global longitudinal strain (GLS)	15.99 ± 3.76	16.49 ± 3.52	14.35 ± 4.07	< 0.001
Tricuspid annular plane systolic excursion (TAPSE)	2.13 ± 0.44	2.19 ± 0.42	1.92 ± 0.45	< 0.001
Tricuspid annulus systolic velocity (Sʹ), cm/s	12.05 ± 3.36	12.69 ± 3.17	10.15 ± 3.17	< 0.001
Abnormal tricuspid annulus systolic velocity, (< 9.5 cm/s)	141 (19.5)	64 (11.9)	77 (41.4)	< 0.001
Free-wall right ventricular strain	20.08 ± 6.81	21.18 ± 6.39	16.85 ± 7.03	< 0.001
Total right ventricular strain	17.15 ± 5.58	18.13 ± 5.28	14.34 ± 5.51	< 0.001
Left atrial reservoir strain	31.54 ± 16.38	34.34 ± 16.03	23.03 ± 14.45	< 0.001
Left atrial conduit strain	21.39 ± 11.52	22.02 ± 11.82	19.16 ± 10.27	0.382
Left atrial pump	12.82 ± 7.25	13.29 ± 7.22	11.16 ± 7.25	0.165
**Tricuspid regurgitation**				0.495
None-trace	549 (75.8)	410 (76.4)	139 (74.2)	
Mild	143 (19.8)	106 (19.7)	37 (19.9)	
Mild-moderate	18 (2.5)	13 (2.4)	5 (2.7)	
Moderate-severe	14 (1.9)	8 (1.5)	6 (3.2)	
Right atrial pressure, mmHg	7.41 ± 3.69	7.24 ± 3.24	7.88 ± 4.71	0.925
PA systolic pressure, mmHg	37.85 ± 13.13	37.65 ± 13.20	38.41 ± 13.00	0.532
Early diastolic mitral vel. (E), cm/s	74.99 ± 22.83	72.37 ± 20.21	82.69 ± 27.89	< 0.001
Late diastolic mitral vel. (A), cm/s	74.87 ± 23.21	73.19 ± 21.65	81.26 ± 27.54	< 0.001
Mitral E/A ratio	1.07 ± 0.47	1.06 ± 0.42	1.09 ± 0.65	0.158
(Septal-eʹ), cm/s	6.73 ± 2.10	7.03 ± 2.06	5.85 ± 1.97	< 0.001
(Lateral-e'), cm/s	8.65 ± 2.63	8.92 ± 2.54	7.86 ± 2.75	< 0.001
Average-eʹ, cm/s	7.69 ± 2.21	7.98 ± 2.15	6.86 ± 2.17	< 0.001
Average E/eʹ ratio	10.75 ± 5.50	9.72 ± 3.84	13.83 ± 7.99	< 0.001
Septal E/eʹ ratio	12.44 ± 6.73	11.10 ± 4.76	16.37 ± 9.57	< 0.001
Lateral E/eʹ ratio	9.71 ± 5.29	8.80 ± 3.69	12.42 ± 7.81	< 0.001

Mean ± standard deviation or count (column%) reported.

*PA* pulmonary artery, *vel.* Velocity.

### Clinical outcomes

The average duration of hospitalization was 13 days (range; 6–24 days) and 425 patients (59%) were admitted to the ICU. Patients admitted to ICU had a significantly higher incidence of diabetes mellitus, respiratory disease, and kidney disease (all P < 0.001). Likewise, serum levels of AST, LDH, CrP (C reactive protein), IL-6, and ferritin were significantly higher (all P < 0.0010) in patients admitted to ICU compared to those who were not admitted to ICU. Troponin was borderline higher in patients admitted to ICU (all P = 0.049). There were no statistically significant differences in echocardiographic measurements between patients admitted to the ICU and those who were not admitted to ICU.

There were 349 patients (48%) who needed mechanical ventilation. Patients who were placed on mechanical ventilation had a significantly higher incidence of diabetes mellitus, respiratory disease, and kidney disease (all P < 0.001). Likewise, serum levels of AST, LDH, CrP, IL-6, and ferritin were significantly higher (all P < 0.0010) in patients on mechanical ventilation compared to those did not need mechanical ventilation. Forty patients were placed on ECMO support. There were 79 total deaths (10.9%).

### Modeling for outcome measures

**Figure 1:**
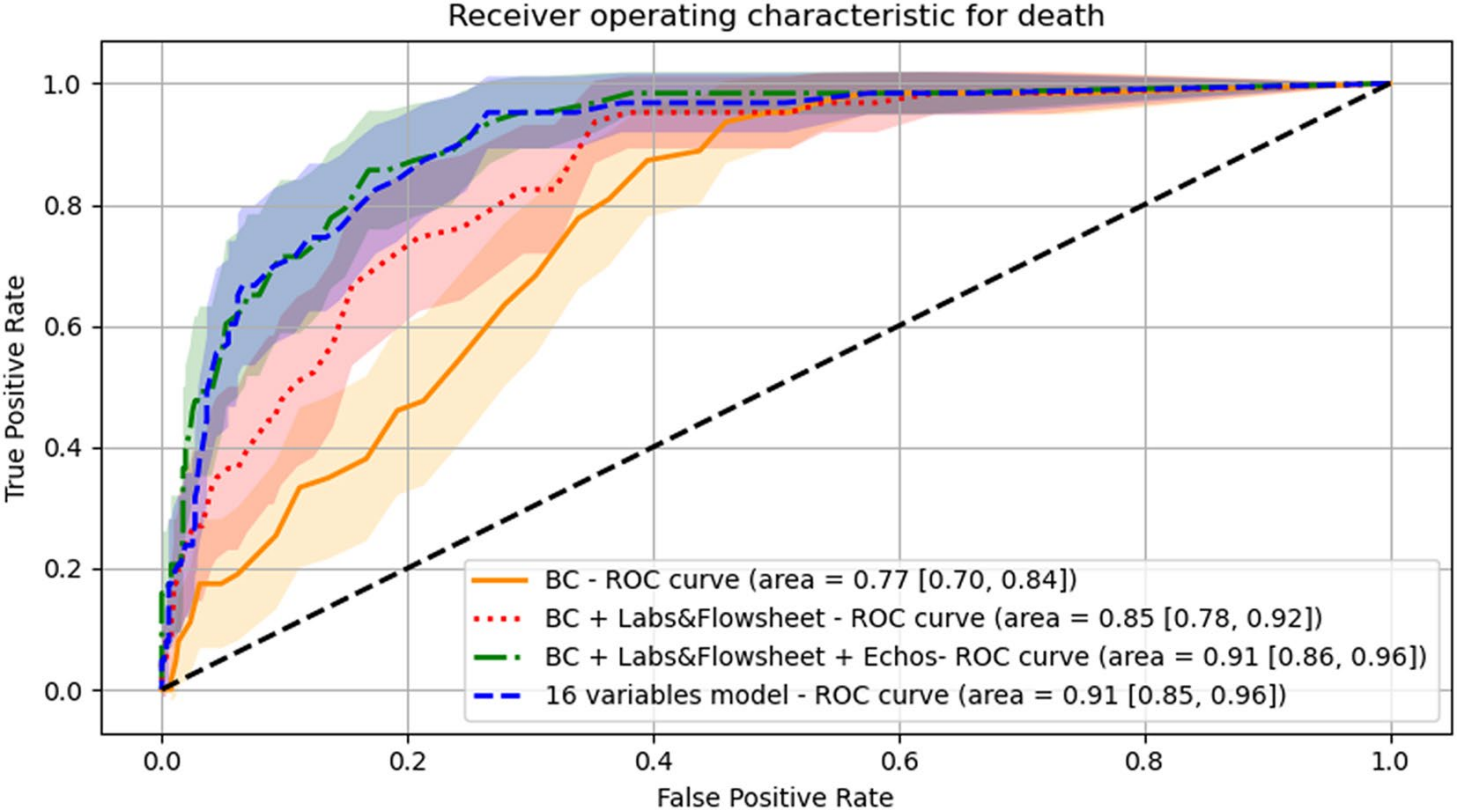
Receiver operator characteristic (ROC) curve for the prediction of death in the study cohort. AUC for the model using baseline characteristics (BC) is shown in orange and yellow for confidence intervals. AUC for the model using baseline characteristics in addition to laboratory data and vital signs (flowsheet) is shown in red and pink for confidence intervals. AUC for the model using baseline characteristics + laboratory data and vital signs (flowsheet) + echocardiographic measurements is shown in blue and shades of blue for confidence intervals. AUC for the optimized prediction model using 16 variables encompassing data from baseline characteristics, vital signs, and echocardiographic measurements is shown in green and shades of green for the confidence intervals.

**Figure 2:**
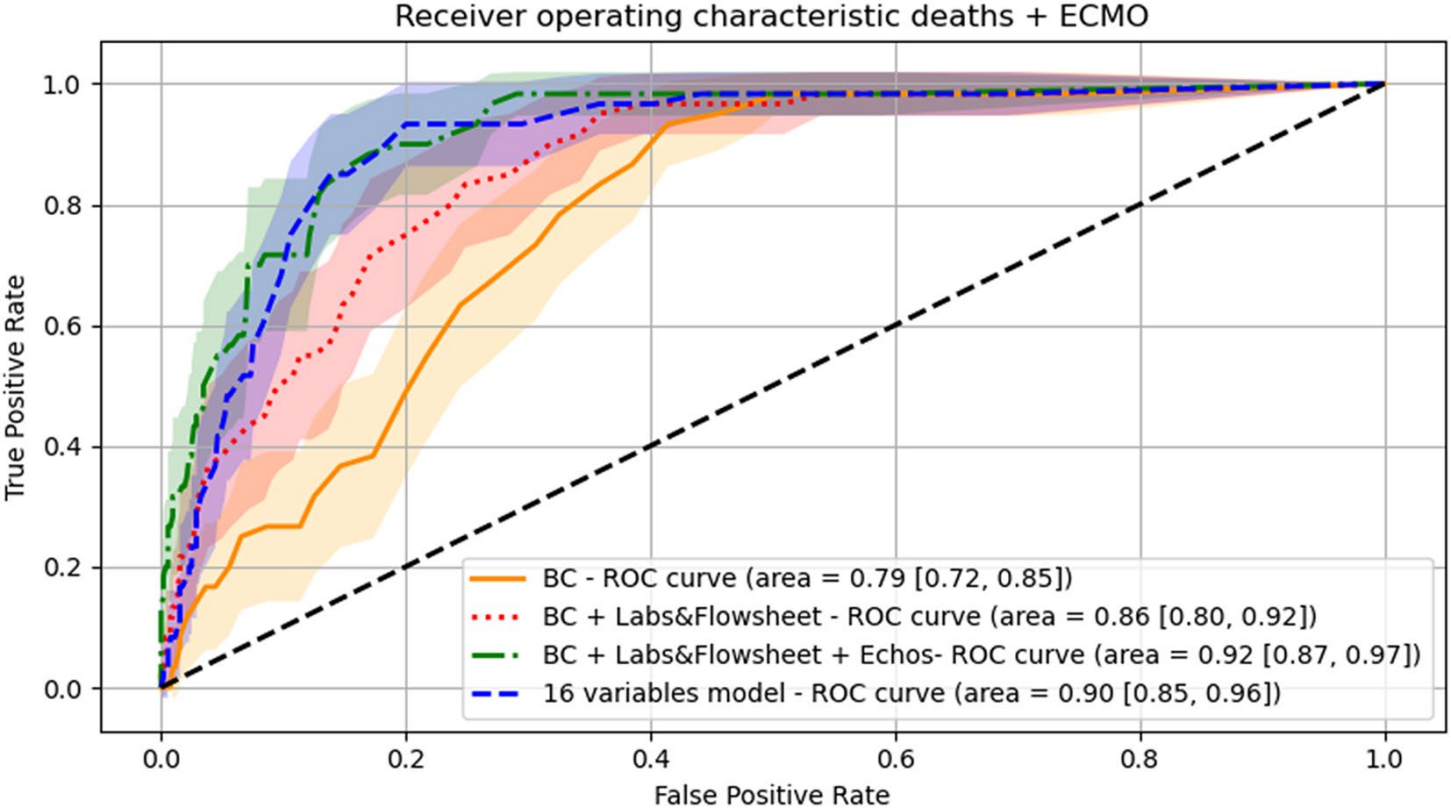
Receiver operator characteristic (ROC) curve for the prediction of death and extracorporeal membrane oxygenation (ECMO) in the study cohort. AUC for the model using baseline characteristics is shown in orange and yellow for confidence intervals. AUC for the model using baseline characteristics in addition to laboratory data and vital signs (flowsheet) is shown in red and pink for confidence intervals. AUC for the model using baseline characteristics + laboratory data and vital signs (flowsheet) + echocardiographic measurements is shown in blue and shades of blue for confidence intervals. AUC for the optimized prediction model using 16 variables encompassing data from baseline characteristics, vital signs, and echocardiographic measurements is shown in green and shades of green for the confidence intervals.

The optimized prediction model included age, body mass index, presence of respiratory disease, presence of renal disease, BNP, IL-6, C reactive protein, ferritin, stroke volume index, free-wall right ventricular strain, total right ventricular strain, left atrial pump strain, LV GLS, right atrial pressure, pulmonary artery systolic pressure, and average E/eʹ ratio. Figure 1 shows the ROC curves for the 3 logistic regression models and the optimized prediction model combining the above variables. A stepwise increase in AUC was observed with the addition of vital signs and laboratory measurements to baseline clinical characteristics, and a further increase (to an AUC 0.91) was observed when echocardiographic measurements were added. The performance of the optimized prediction model was similar to the model including baseline characteristics + vital signs and laboratory results + echocardiographic measurements. Figure 2 shows the same approach but for the prediction of the combined end point of deaths and ECMO.

## Discussion

In patients without previous cardiovascular disease, EF < 50% occurred in 3.4%, abnormal GLS (< 16%) was observed in 24%, and LV diastolic dysfunction in 20%. RV systolic dysfunction defined by RV free wall strain < 20% was noted in 16%. Moderate and large pericardial effusion were uncommon with an incidence of 0.4% for each category.

Previous studies reported a variable incidence of abnormal LV systolic function, based on the criteria used to define systolic dysfunction and whether patients with known cardiac disease were excluded. Expectedly, the incidence is higher if the more sensitive index of LV systolic function, GLS, is used to detect disease. An abnormal LV EF was much less common and was mostly due to global dysfunction with very few patients having stress induced cardiomyopathy, as noted in previous reports with adult patients^13^. The majority of patients without prior cardiac diagnosis had normal estimates of left atrial pressure but in 6%, findings were consistent with elevated left atrial pressure. Therefore, it appears most patients had shortness of breath due to non-cardiac etiologies. This information is of value in directing diuretic therapy to patients with evidence of increased left atrial pressure. The apparent higher incidence of reduced GLS than LV diastolic dysfunction may be accounted for by the 5% of patients where diastolic function status could not be ascertained based on the available data gathered. While acute viral infection and the host immune response can explain the abnormal LV function, there are competing reasons for the abnormal LV function aside from the acute COVID-19 illness as 52% were obese, 52% had diabetes, and 62% had kidney disease, all of which can cause abnormal myocardial function including diastolic dysfunction.

RV function as assessed by echocardiographic indices is affected not only by intrinsic RV contractility but also by loading conditions. In the setting of pulmonary disease due to COVID-19 infection, hypoxemia can develop along with pulmonary vasoconstriction leading to RV systolic dysfunction in the absence of primary myocardial disease. Treatment of pulmonary parenchymal and vascular pathology would therefore be expected to have a favorable effect on RV function, to the extent that these treatments are effective. Notwithstanding the underlying mechanisms for RV dysfunction, RV systolic dysfunction is associated with worse clinical outcomes^1–3,5,8,15,18,19,22,23^. In comparison with previous studies, the incidence of RV dysfunction was lower in our cohort but when present, it was still associated with worse outcome.

Several of COVID-19 patients who are hospitalized have an increased risk of adverse events. It is of value to identify those at higher risk so as to consider different management algorithms. In approaching the patients in this study sample, we looked at baseline demographics and clinical characteristics and used these data as the first level with which to compare the incremental value of other variables since they represent the initial set of data available to the treating physicians. Similar to previous findings, older patients with higher body mass index and those with respiratory or renal disease were at higher risk of death. With respect to the second group of laboratory findings, BNP, IL-6, C reactive protein, and ferritin added to the baseline risk as AUC significantly increased from 0.77 to 0.85. This by itself is useful to consider even in patients who have not undergone echocardiographic imaging. While routine imaging in all patients is not recommend, the incremental value of data on biventricular function when there is an appropriate echocardiographic indication is evident as AUC further increased with the addition of echocardiographic variables from 0.85 to 0.91. Further, the optimized prediction model with AUC at 0.91 included stroke volume index, RV free wall strain, total RV strain, left atrial pump strain, LV GLS, right atrial pressure, pulmonary artery systolic pressure, and average E/e’ ratio (a marker of left atrial pressure).

One of the 3 indications for obtaining an echocardiogram was the abnormal elevation in troponin levels. Troponin elevation is influenced by baseline characteristics including preexisting cardiac disease^29–31^. Troponin elevation may be misleading with respect to drawing the conclusion of myocardial disease being due to COVID-10 infection, since coronary syndromes can also cause this finding. On the other hand, echocardiographic imaging can provide more specific evidence of coronary disease as well as a detailed look at the extent of cardiac pathology in the setting of acute COVID-19 infection.

The findings are applicable to hospitalized COVID-19 patients and may not apply to patients whose clinical status does not warrant admission. Echocardiography was not obtained in all hospitalized patients as only those who had a clinical indication were scanned. This could have led to an overestimation of the incidence of cardiac abnormalities. Notwithstanding, the study findings are applicable to the current practice as routine imaging is not obtained in all patients in the absence of an appropriate indication.

There were no cardiac biopsies obtained, and thus it is difficult to determine if abnormal LV function is due to viral infection of cardiomyocytes, cytokines induced cardiac dysfunction, or is the consequence of adverse effects of hypoxemia in some patients. CMR (cardiac magnetic resonance) was not obtained in these patients in the acute setting based on judgement of the treating physicians but could have shed light about the possible presence of replacement fibrosis, increased extracellular volume, and tissue edema. While a large number of patients was included in this study, additional multicenter studies with many more patients can provide other insights. We did not include a control group of patients with similar baseline characteristics but without viral infection. However, the summary statistics for the echocardiographic measurements in this study are very similar to other studies which included a control group^15^.

## Data Availability

The datasets generated during and/or analyzed during the current study are available from the corresponding author on reasonable request.
